# Carcass composition and physicochemical and sensory attributes of breast and leg meat from native Mexican guajolote (*Meleagris g. gallopavo*) as influenced by sex

**DOI:** 10.5194/aab-66-341-2023

**Published:** 2023-11-20

**Authors:** Rodrigo Portillo-Salgado, José Guadalupe Herrera-Haro, Jaime Bautista-Ortega, Jacinto Efrén Ramírez-Bribiesca, Carolina Flota-Bañuelos, Alfonso Juventino Chay-Canul, Francisco Antonio Cigarroa-Vázquez

**Affiliations:** 1 Tecnológico Nacional de México, Instituto Tecnológico Superior de Calkiní, 24206 Calkiní, Campeche, Mexico; 2 Colegio de Postgraduados, Campus Montecillo, 56230 Montecillo, Estado de México, Mexico; 3 Colegio de Postgraduados, Campus Campeche, 24450 Champotón, Campeche, Mexico; 4 CONACYT, Colegio de Postgraduados, Campus Campeche, 24450 Champotón, Campeche, Mexico; 5 División Académica de Ciencias Agropecuarias, Universidad Juárez Autónoma de Tabasco, 86280 Villahermosa, Tabasco, Mexico; 6 Escuela de Estudios Agropecuarios Mezcalapa, Universidad Autónoma de Chiapas, 29622 Copainalá, Chiapas, Mexico

## Abstract

The aim of the study was to compare carcass composition and physicochemical and sensory attributes of breast and leg meat from native Mexican guajolote (*Meleagris g. gallopavo*) as influenced by sex. For this, slaughter weight and carcass characteristics of male ( n=8 ) and female ( n=8 ) guajolotes raised traditionally under extensive systems with similar housing and feeding conditions were recorded. Also, physical characteristics, proximate composition, the fatty acid profile, and sensory attributes were determined in breast and leg meat using standard procedures. The results showed that males had higher ( P<0.001 ) slaughter weight, hot and cold carcass weights, and dressing percentage, as well as carcass part weights, while females had higher ( P<0.001 ) abdominal fat weights than males. The lightness ( $L^{*}$ ), yellowness ( $b^{*}$ ), and drip loss values of breast meat, as well as redness ( $a^{*}$ ) and water-holding capacity values of leg meat, were significantly ( P<0.05 ) influenced by sex. Male breast meat had higher ( P<0.05 ) moisture content, crude protein, erucic acid (C22:1n9), ∑ MUFAs (total monounsaturated fatty acids), ∑ UFAs (unsaturated fatty acids), ∑ DFAs (desirable fatty acids), ∑ UFA / ∑ SFA (total saturated fatty acid) ratio, ∑ PUFA (total polyunsaturated fatty acid) / ∑ SFA ratio, and chewiness scores than females. Likewise, leg meat from males showed higher ( P<0.05 ) ash content, myristic acid (C14:0), palmitic acid (C16:0), stearic acid (C18:0), oleic acid (C18:1n9c), palmitoleic acid (C16:1n7), ∑ SFAs, ∑ OFAs (odd fatty acids), thrombogenic index, and atherogenic index, whereas females had high fat content. In conclusion, it would be suggested that, from a nutritional point of view, the meat from male guajolotes was preferable to the meat from females.

## 1 Introduction

The poultry sector in developing countries is largely based on traditional production or free-range (extensive) systems that are characterized by low input and more limited production outputs (Manyelo et al., 2020). In these poultry production systems, native or local poultry breeds are mainly used, and they play a substantial role for the rural poor and marginalized section of the people with respect to their subsidiary income and food security, since they provide them with meat and egg for consumption and sale (Padhi, 2016; Pius et al., 2021; Kanakachari et al., 2022). These poultry genotypes (e.g., Aseel, Fulani, Busra, Deshi, Naked neck, Nicobari, frizzle, Punjab Brown, Thai native, Tepi, Hilly, Matrouh, Mandarah, and Fayoumi) are well known for their desirable biological characteristics, such as good adaptability, thermotolerance, and resistance to diseases (Padhi, 2016; Mengesha et al., 2022). In addition, their meat is considered to have a desirable taste and flavor; therefore, there is growing interest among poultry farmers and meat consumers in native germplasm because of its unique characteristics (Rajkumar et al., 2016).

In recent years, there has been a new trend in poultry meat consumption, with a strong demand for meat from free-range or organic systems, which ensures food security and animal welfare combined with environmental responsibility, consumer health, and better meat quality (Özbek et al., 2020). Poultry meat quality can be observed by its nutritive value and sensory characteristics. Poultry meat is an essential source of food due to its favorable effects on human health derived from its protein, fats, minerals, vitamins, and bioactive components (Attia et al., 2017). Sensory attributes include meat color, aroma, texture, and flavor and are important factors that determine consumer preference for a product (Uhlířová et al., 2018). Consumers often seek meat that is low in fat, tender, and juicy with a good aroma and flavor (Selamat et al., 2022). However, carcass characteristics and meat quality in poultry may be influenced by several factors such as sex, breed, origin, weight and age at slaughtering, feeding, breeding, management (pre-slaughter, stunning, slaughter and post-slaughter procedures, chilling, and storage conditions), and environmental factors (Onk et al., 2019; El-Tarabany et al., 2022; González Ariza et al., 2022). On the other hand, carcass quality is also determined by the distribution of tissue components. Lean meat should be located in the most valuable carcass parts (breast and legs). The tissue composition of poultry carcasses changes with age because the growth rates of tissue components vary across gender and species (Murawska, 2017).

The native guajolote (*Meleagris g. gallopavo*) is the second most important poultry species in Mexico, after chicken (Romero-López, 2021), and is known to be the genetic base of the breeds and varieties of turkeys that are known nowadays (Portillo-Salgado et al., 2022a). According to the Agri-Food and Fisheries Information Service (Servicio de Información Agroalimentaria y Pesquera – SIAP, 2021), in 2021, the total chicken population in Mexico (including laying hens and chicken for meat) was 604.68 million birds, while the total population of guajolotes was only 3.79 million birds. The native guajolote has more genetic variation than the commercial turkey due to genetic isolation and a longer period of genetic adaptation to local environmental conditions but is less studied than the commercial turkey (Camacho-Escobar et al., 2008). In this context, the native guajolote is noted for its good adaptability and high rusticity that allows it to reproduce under different environmental and management conditions. Also, it has a good capacity to convert feed into meat due to its good muscle development (Portillo-Salgado et al., 2022a). It is raised mainly in rural and sub-urban regions, under backyard conditions or extensive systems based on grazing and limited use of inputs. Their products (meat and eggs) constitute a main source of protein and income, being a source of investment and security for rural households (Portillo-Salgado et al., 2022b). Guajolote meat is considered one of the healthiest, characterized for having little fat and a low cholesterol level (Portillo-Salgado et al., 2022a). An important attribute given by consumers to guajolote meat is that this meat has a better taste than that of the commercial turkey (Ramírez-Rivera et al., 2012). However, the suitability of this native poultry species for niche poultry markets has not been well researched with regard to carcass composition, meat quality, nutritional content, and sensory acceptability. Therefore, the aim of this study was to compare carcass composition and physicochemical and sensory attributes of breast and leg meat from native Mexican guajolote (*Meleagris g. gallopavo*) as influenced by sex.

## 2 Materials and methods

### 2.1 Location, birds, and experimental design

All experimental procedures were conducted at the Laboratory of Animal Science of the Colegio de Postgraduados, Campus Campeche (Campeche, Mexico). For the study, a sample of 16 native guajolotes (male, n = 8 and female, n=8 ) was used. The birds were aged between 10 and 12 months. The main criterion for the selection of birds was average body weight, commonly used by local producers in marketing of this poultry species (5 and 3 kg for male and female, respectively). All birds were purchased from local poultry farms located in the municipalities of Champotón (19 ${}^{\circ }$ 21 ${}^{\prime }$ N, 90 ${}^{\circ }$ 43 ${}^{\prime }$ E; 10 m a.s.l.) and Hopelchén (19 ${}^{\circ }$ 44 ${}^{\prime }$ N, 89 ${}^{\circ }$ 50 ${}^{\prime }$ E; 89 m a.s.l.), where they were raised traditionally under extensive systems with similar housing and feeding conditions (Portillo-Salgado et al., 2018). All birds were dewormed and vaccinated upon arrival. A 15 d adaptation period was applied in order to get the birds used to the new shelter and feeding environment. Native guajolotes were housed per sex in two poultry houses (20 m ${}^{2}$ each) with walls and floor made of concrete equipped with feeders and drinkers and had outdoor access during the day to a grazing area which also contained fruit trees, bushes, and hedges. Birds were confined in the poultry house during the night. The bird's diet was based on inputs that they collect during grazing in the pasture area, such as grasses, plants, seeds, fruits, and insects. Additionally, they received other complementary feeds such as corn, corn dough, wheat bran (*salvadillo*), and kitchen waste (corn tortilla, bread, fruits, and vegetables). Birds had free access to clean water. The study region is characterized by a warm, sub-humid climate with summer rainfall A ( w ); it presents temperatures that oscillate between 18 and 30 ${}^{\circ }$ C and a total annual precipitation of 1600 mm.

### 2.2 Slaughter and carcass traits

Before slaughtering, the birds were subjected to feed withdrawal for 10 h; however, clean drinking water was provided ad libitum during this feed withdrawal period. All birds were weighed and manually slaughtered by exsanguination following the Official Mexican Standards (NOM-008-ZOO-1994, NOM-009-ZOO-1994, and NOM-033-ZOO-1995) established for the humane slaughter of animals intended for meat production. After bleeding for 2 min, the carcasses were scalded in a water bath between 60–65 ${}^{\circ }$ C for 2 min to facilitate manual plucking. Subsequently, neck, head, feet, edible internal organs (heart, liver, and gizzard), and abdominal fat were removed and weighed using an electronic balance ( ± 1 g). Carcasses were weighed to obtain the hot carcass weights. Cold carcass weights were determined after carcasses had been stored at + 4 ${}^{\circ }$ C for 24 h. Dressing percentage was calculated as the percentage of cold carcass weight from slaughter weight. Carcasses were dissected into breast, thigh, drumstick, wings, and back as described by Hahn and Spindler (2002). The carcass parts were weighed using an electronic balance, and their yields were calculated as a percentage of cold carcass weight. Three right breast (*Pectoralis major* and* Pectoralis minor*) and leg (including drumstick and thigh) muscles for each sex were individually vacuum-packed and stored during 30 d at - 20 ${}^{\circ }$ C for descriptive sensory analysis.

### 2.3 Physical and chemical analysis

Before dissection (24 h post mortem), pH value and color parameters of breast (*Pectoralis major*) and leg muscles, without skin, were measured. The pH ${}_{24\;h}$ value was measured with a portable digital pH meter (Model HI 99161, Hanna Instruments^®^, USA), equipped with a glass electrode, which was introduced to a depth of 1 cm in the cross-section of muscle. Before measurement, the pH meter was calibrated using buffers of pH 4.0 and pH 7.0 at room temperature according to the manufacturer's instructions. The pH was evaluated at three points within the muscle, adopting the average value of these three readings. The color parameters were measured using a colorimeter (Model CR-400, Konica Minolta^®^, Tokyo, Japan) and were expressed in terms of CIELab color coordinates reporting values for lightness ( $L^{*}$ ; black/white), redness ( $a^{*}$ ; green/red), and yellowness ( $b^{*}$ ; blue/yellow). The average value of three repeated readings recorded from different points on the surface of the muscles was used.

Water-holding capacity (WHC; %) was evaluated using the filter paper press method (Grau and Hamm, 1953) modified by Biesek et al. (2021). Ground meat samples (3 g) were placed between two sheets of filter paper (Whatman^®^ No. 1). The set was pressed with a standard weight of 2 kg for 5 min. The samples were then removed from the filter paper and weighed. WHC was calculated as the difference between the initial sample weight and the final weight. Cooking loss (CL; %) was determined by placing ground meat samples (20 g) on a absorbent gauze inside sealed plastic bags, and cooked in a water bath at 85 ${}^{\circ }$ C for 10 min (Kokoszyński et al., 2020). Cooked meat samples were chilled at + 4 ${}^{\circ }$ C for 30 min and dried with paper towels. CL was expressed as the ratio between the weight before and after cooking. Drip loss (DL; %) was determined by placing ground meat samples (20 g) in two sealable bags (one of the bags was perforated to allow dripping) and storing them at + 4 ${}^{\circ }$ C for 24 h (Kokoszyński et al., 2020). DL was expressed as the percentage of weight loss of the sample relative to its weight recorded before the refrigeration period.

The proximate composition of breast and leg meat was analyzed according to the methods described by the Association of Official Analytical Chemists (AOAC, 1990). The moisture content (%) of the meat was determined by freeze-drying using a freeze dryer (Labconco^®^, Kansas City, USA). The total crude protein content (%) was obtained according to the AOAC 990.03 Dumas combustion method (AOAC, 2005), while the crude fat content (%) was obtained by the AOAC 991.36 Soxtec submersion method (Thiex et al., 2003). Finally, the ash content (%) was analyzed by incineration at 600 ${}^{\circ }$ C for 2 h according to the AOAC 942.05 method (Thiex and Novotny, 2012).

### 2.4 Fatty acid analysis

The fatty acids' profile was determined from one pool per muscle type and per sex by gas chromatography following the methods of AOAC Official Method 996.06 (Analysis of Methyl Esters by Capillary GLC) and AOCS Official Method Ce 2–66 (Preparation of Methyl Esters of Fatty Acids). A total of four pools were formed (one pool of breast meat per sex and one pool of leg meat per gender). Each pool comprised 12 g lyophilized meat (2 g per bird).

The average amount of each fatty acid was used to calculate the sum of the total saturated ( ∑ SFAs = C12:0 + C14:0 + C16:0 + C18:0 + C20:0 + C24:0), total monounsaturated ( ∑ MUFAs = C16:1n7 + C18:1n9c + C22:1n9), and total polyunsaturated ( ∑ PUFAs = C18:2n-6c) fatty acids. Unsaturated fatty acids (UFAs) were the sum of MUFAs and PUFAs. Desirable fatty acids (DFAs) were C18:0 and UFAs. Odd fatty acids (OFAs) were the sum of C14:0 and C16:0 (Belhaj et al., 2020). Nutritional indices of lipids were calculated as follows: thrombogenic index (TI) = (C14:0 + C16:0 + C18:0) / [(0.5 × ∑ MUFAs) + (0.5 × ∑ *n-6*) + (3 × ∑ *n-3*) + ( ∑ *n-3/* ∑ *n-6*)], atherogenic index (AI) = (C12:0 + 4 × C14:0 + C16:0) / ∑ UFAs (Ulbricht and Southgate, 1991), and nutritive value index (NVI) = (C18:0 + C18:1) / C16:0 (Chen et al., 2016).

### 2.5 Descriptive sensory evaluation

The frozen breast and leg muscles were thawed for 24 h at 4 ${}^{\circ }$ C (Amorin et al., 2016). Later, the muscles were deboned to obtain several meat fillets, which were tagged with codes and cooked in boiling water for 30 min at 100 ${}^{\circ }$ C until they reached a core temperature of 76 ${}^{\circ }$ C, without adding salt or seasoning (Ramírez-Rivera et al., 2012). The samples were prepared following appropriate food handling practices (Toomer et al., 2019). All fillets were cooked in the same amount of water, at the same temperature, and for the same time duration. The core temperature was determined using a digital meat thermometer, inserted after removing the cooked meat fillets from hot water. Cooked meat fillets were allowed to cool for 20 min and then cut into cubes of 1 × 1 × 1 cm. Subsequently, meat portions were placed in aluminum pans and covered with aluminum foil. To ensure meat sample quality, they were kept in an oven at a constant temperature (75–80 ${}^{\circ }$ C) throughout the sensory test time.

For sensory evaluation, a panel of 20 individuals (15 males and 5 females with ages between 23 and 62 years) from the academic and student population of Colegio de Postgraduados (Campeche, Mexico) was selected. All panelists had previous experience in the consumption of poultry meat. Each panelist received a set of four meat samples (one sample by each sex and muscle type), as well as the list of sensory attributes evaluated. There was a 5 min interval between serving each meat sample. Consumers evaluated various attributes related to consumer taste (flavor, tenderness, chewiness, juiciness), intensity (aroma and color), and overall acceptance using a seven-point hedonic scale (Tan et al., 2022), where 1 denotes “dislike extremely” or low intensity, and 7 denotes “like extremely” or high intensity. The sensory attributes and their description are described by Semwogerere et al. (2019). Consumers were provided with water at room temperature and fresh bread for palate cleansing and to neutralize their sensory percepts (Uhlířová et al., 2018; Toomer et al., 2019).

### 2.6 Statistical analysis

Data collected for carcass composition and meat quality were analyzed using the general linear model (GLM) procedure of the SAS Version 9.4 statistical package (SAS Institute Inc. Cary, NC, USA, 2016). The linear model used for carcass composition traits (carcass characteristics, internal organs and non-carcass components) was $Y_{ij}=\mu +S_{i}+e_{ij}$ , where $Y_{ij}$ is the response variable, μ is the overall mean common to all observations, $S_{i}$ is the effect of i th sex (male and female), $e_{ij}$ is the random error with a mean of 0, and variance is $\sigma^{2}$ , whereas meat quality traits (physicochemical and sensory attributes) were analyzed using the following linear model: $Y_{ijk}=\mu +S_{i}+M_{j}+$ ( S × M ) ${}_{ij}+e_{ijk}$ , where $Y_{ijk}$ is the response variable, μ is the overall mean common to all observations, $S_{i}$ is the effect of i th sex (male and female), $M_{j}$ is the effect of j th muscle type (breast and leg), ( S × M ) ${}_{ij}$ is the sex by muscle type interaction, $e_{ijk}$ is the random error with a mean of 0, and variance is $\sigma^{2}$ . Normal distribution of the variables was analyzed according to the Shapiro–Wilk test. The results are presented as least-square means ± standard error of the mean (SEM). None of the interactions were significant and are therefore not reported in the results. Differences were considered significant at the level of P≤0.05 . For statistical analyses, each bird was considered the experimental unit.

## 3 Results

### 3.1 Carcass characteristics

The results on slaughter weight and carcass characteristics of native Mexican guajolotes are reported in Table 1. Males had higher ( P<0.001 ) slaughter weight, hot and cold carcass weights, and dressing percentage than females. Carcass part weights were heavier ( P<0.001 ) in males, while females had higher drumstick ( P<0.05 ) and wing ( P<0.001 ) yields. The thigh and back yields did not vary significantly by sex ( P>0.05 ). Heart and liver were heavier ( P<0.001 ) in males than in females, although significantly higher ( P<0.00 1) abdominal fat weights were obtained in females. Males had heavier ( P<0.001 ) neck, head, and feet than females.

**Table 1:** Means ( ± standard error) of carcass characteristics, internal organs, and non-carcass components of native Mexican guajolote as influenced by sex.

Item	Male	Female	P value
Carcass characteristics			
Slaughter weight (g)	5688.75 ± 250.11	2913.13 ± 189.73	<0.001
Hot carcass weight (g)	3740.63 ± 224.88	1743.13 ± 104.80	<0.001
Cold carcass weight (g)	3719.38 ± 223.91	1733.75 ± 104.20	<0.001
Dressing percentage ${}^{1}$ (%)	65.43 ± 1.24	59.97 ± 0.61	0.001
Breast (g)	1285.63 ± 107.77	516.90 ± 38.57	<0.001
Thigh (g)	678.12 ± 19.52	322.50 ± 20.89	<0.001
Drumstick (g)	556.87 ± 19.01	298.75 ± 14.41	<0.001
Wings (g)	423.75 ± 8.16	249.38 ± 9.65	<0.001
Back (g)	745.62 ± 83.57	345.63 ± 27.09	0.001
Breast (%) ${}^{2}$	34.20 ± 1.02	29.65 ± 0.53	0.001
Thigh (%) ${}^{2}$	18.60 ± 0.99	18.57 ± 0.15	0.977
Drumstick (%) ${}^{2}$	15.17 ± 0.52	17.36 ± 0.65	0.020
Wings (%) ${}^{2}$	11.62 ± 0.58	14.50 ± 0.33	0.001
Back (%) ${}^{2}$	19.66 ± 1.07	19.85 ± 0.72	0.885
Internal organs			
Heart (g)	26.00 ± 1.45	13.00 ± 1.18	<0.001
Liver (g)	81.37 ± 5.52	52.25 ± 3.43	0.001
Gizzard (g)	75.75 ± 5.54	75.37 ± 4.53	0.959
Abdominal fat (g)	61.71 ± 7.03	175.31 ± 23.74	0.001
Non-carcass components			
Neck (g)	293.75 ± 27.30	106.88 ± 8.50	<0.001
Head (g)	157.50 ± 11.45	89.38 ± 3.19	<0.001
Feet (g)	177.50 ± 4.81	96.25 ± 2.05	<0.001

${}^{1}$ Cold carcass weight/slaughter weight × 100. ${}^{2}$ Calculated as a percentage of the cold carcass weight.

### 3.2 Physical characteristics

The physical attributes of breast and leg meat from native Mexican guajolotes are shown in Table 2. The lightness ( $L^{*}$ ), yellowness ( $b^{*}$ ), and drip loss values of breast meat, as well as redness ( $a^{*}$ ) and water-holding capacity values of leg meat, were significantly ( P<0.05 ) influenced by sex. On the other hand, the breast meat from males was characterized by higher ( P<0.05 ) lightness ( $L^{*}$ ) and water-holding capacity values and lower ( P<0.05 ) redness ( $a^{*}$ ) and yellowness ( $b^{*}$ ) values compared to leg meat. Similarly, in females, the breast meat presented higher ( P<0.05 ) water-holding capacity and drip loss values but lower ( P<0.001 ) pH ${}_{24\;h}$ and redness ( $a^{*}$ ) values than leg meat.

**Table 2:** Means ( ± standard error) of physical characteristics in native Mexican guajolote meat as influenced by sex and muscle type.

Item	Muscle	Sex		P value
		Male	Female	
pH ${}_{24\;h}$	Breast	5.81 ± 0.03	5.75 ± 0.03	0.301
	Leg	5.90 ± 0.03	6.00 ± 0.03	0.054
	P value	0.078	0.001	
Color ${}_{24\;h}$				
$L^{*}$	Breast	39.57 ± 1.84	27.54 ± 2.85	0.003
	Leg	28.13 ± 1.73	28.73 ± 1.97	0.824
	P value	0.001	0.738	
$a^{*}$	Breast	1.49 ± 0.38	1.55 ± 0.21	0.894
	Leg	9.65 ± 0.37	8.07 ± 0.64	0.048
	P value	<0.001	<0.001	
$b^{*}$	Breast	4.89 ± 0.59	9.00 ± 1.12	0.005
	Leg	8.36 ± 0.91	9.65 ± 0.55	0.264
	P value	0.006	0.611	
Water-holding capacity (%)	Breast	64.62 ± 2.29	56.25 ± 4.21	0.102
	Leg	55.33 ± 2.94	42.75 ± 2.03	0.003
	P value	0.026	0.012	
Cooking loss (%)	Breast	21.48 ± 1.40	21.12 ± 0.76	0.824
	Leg	21.72 ± 2.21	22.07 ± 1.78	0.904
	P value	0.929	0.633	
Drip loss (%)	Breast	2.70 ± 0.40	3.96 ± 0.20	0.014
	Leg	2.81 ± 0.25	2.82 ± 0.21	0.970
	P value	0.817	0.001	

### 3.3 Proximate composition

The chemical composition of breast and leg meat from native Mexican guajolotes is presented in Table 3. Male breast meat had higher ( P<0.05 ) moisture content and crude protein but lower ash content than that of females. Regarding leg meat, males showed higher ( P<0.05 ) ash content, whereas females had high fat content. In addition, it was observed that in both sexes, the moisture and fat contents were higher ( P<0.001 ) in leg meat than in breast meat. However, breast meat had higher ( P<0.001 ) crude protein values than leg meat. In females, breast meat had higher ( P<0.001 ) ash content than leg meat.

**Table 3:** Means ( ± standard error) of chemical composition in native Mexican guajolote meat as influenced by sex and muscle type.

Item	Muscle	Sex		P value
		Male	Female	
Moisture content (%)	Breast	73.95 ± 0.16	72.98 ± 0.24	0.005
	Leg	74.98 ± 0.14	74.45 ± 0.24	0.080
	P value	0.001	0.001	
Crude protein (%)	Breast	24.19 ± 0.24	22.62 ± 0.22	0.003
	Leg	20.42 ± 0.14	20.31 ± 0.07	0.512
	P value	<0.001	<0.001	
Fat (%)	Breast	0.97 ± 0.13	1.51 ± 0.25	0.079
	Leg	2.01 ± 0.09	3.06 ± 0.15	<0.001
	P value	<0.001	0.001	
Ash (%)	Breast	1.09 ± 0.00	1.12 ± 0.00	0.015
	Leg	1.12 ± 0.01	1.08 ± 0.01	0.016
	P value	0.073	0.003	

### 3.4 Fatty acid profile

The composition of individual fatty acids and the nutritive indices of breast and leg meat from native Mexican guajolotes are described in Tables 4 and 5. Male breast meat had higher ( P<0.05 ) proportions of erucic acid (C22:1n9), ∑ MUFAs, ∑ UFAs, ∑ DFAs, ∑ UFA / ∑ SFA ratio, and ∑ PUFA / ∑ SFA ratio than females. In contrast, the proportions of arachidonic acid (C20:0), ∑ SFAs, and ∑ OFAs were higher in breast meat of females than that of males. Meanwhile, leg meat of males presented a higher ( P<0.05 ) content of erucic acid (C22:1n9), ∑ UFAs, and ∑ DFAs but a lower content of arachidonic acid (C20:0), ∑ SFAs, ∑ OFAs, and atherogenic index (AI) than leg meat of females.

**Table 4:** Means ( ± standard error) of fatty acids (g per 100 g of fat) in native Mexican guajolote meat as influenced by sex and muscle type.

Item	Muscle	Sex		P value
		Male	Female	
Lauric acid (C12:0)	Breast	0.60 ± 0.00	3.70 ± 1.60	0.467
	Leg	0.60 ± 0.10	5.05 ± 1.58	0.175
	P value	1.000	0.582	
Myristic acid (C14:0)	Breast	0.95 ± 0.04	3.22 ± 1.55	0.165
	Leg	1.52 ± 0.09	2.50 ± 0.43	0.061
	P value	<0.001	0.660	
Palmitic acid (C16:0)	Breast	12.20 ± 0.24	13.03 ± 0.74	0.302
	Leg	15.35 ± 0.43	16.40 ± 0.64	0.196
	P value	<0.001	0.004	
Palmitoleic acid (C16:1n7)	Breast	5.96 ± 0.46	6.51 ± 1.15	0.665
	Leg	7.33 ± 0.29	7.40 ± 0.39	0.901
	P value	0.024	0.480	
Stearic acid (C18:0)	Breast	9.08 ± 0.28	12.01 ± 2.90	0.332
	Leg	11.86 ± 0.47	11.12 ± 0.58	0.343
	P value	0.001	0.768	
Oleic acid (C18:1n9c)	Breast	25.53 ± 0.44	23.35 ± 2.93	0.472
	Leg	31.33 ± 0.77	32.78 ± 1.34	0.366
	P value	<0.001	0.011	
Linoleic acid (C18:2n-6c)	Breast	34.87 ± 0.74	31.13 ± 4.54	0.430
	Leg	28.27 ± 1.07	23.85 ± 2.01	0.073
	P value	0.001	0.164	
Arachidonic acid (C20:0)	Breast	1.00 ± 0.11	2.83 ± 0.39	0.001
	Leg	0.56 ± 0.04	1.40 ± 0.30	0.021
	P value	0.008	0.018	
Erucic acid (C22:1n9)	Breast	9.11 ± 1.25	5.32 ± 0.88	0.027
	Leg	3.78 ± 0.25	1.95 ± 0.67	0.018
	P value	0.003	0.032	
Lignoceric acid (C24:0)	Breast	1.15 ± 0.43	0.70 ± 0.05	0.560
	Leg	1.50 ± 0.62	0.35 ± 0.25	0.356
	P value	0.645	0.179	

On the other hand, the fatty acid profiles were significantly different among muscle types. In males, the proportions of myristic acid (C14:0), palmitic acid (C16:0), stearic acid (C18:0), oleic acid (C18:1n9c), palmitoleic acid (C16:1n7), ∑ SFAs, ∑ OFAs, thrombogenic index (TI), and atherogenic index (AI) were found to be higher ( P<0.05 ) in leg meat than in breast meat. However, the results demonstrated the highest content of arachidonic acid (C20:0), erucic acid (C22:1n9), ∑ PUFAs, ∑ UFAs, ∑ DFAs, ∑ UFA / ∑ SFA ratio, and ∑ PUFA / ∑ SFA ratio in breast meat when compared to leg meat. In females, the concentration of arachidonic acid (C20:0), and erucic acid (C22:1n9) was higher ( P<0.05 ) in breast meat than in leg meat. Conversely, leg meat was characterized by a higher ( P<0.05 ) proportion of palmitic acid (C16:0), oleic acid (C18:1n9c), and ∑ MUFAs than breast meat. Palmitic acid (C16:0), oleic acid (C18:1n9c), and linoleic acid (C18:2n-6c) were the most abundant SFAs, MUFAs, and PUFAs, respectively.

**Table 5:** Means ( ± standard error) of nutritional indices of the lipids in native Mexican guajolote meat as influenced by sex and muscle type.

Item	Muscle	Sex		P value
		Male	Female	
∑ SFAs	Breast	24.46 ± 0.54	33.72 ± 4.17	0.045
	Leg	30.25 ± 0.79	34.95 ± 1.97	0.044
	P value	<0.001	0.794	
∑ MUFAs	Breast	40.61 ± 0.87	35.18 ± 1.93	0.022
	Leg	41.51 ± 1.06	41.16 ± 1.50	0.852
	P value	0.523	0.028	
∑ PUFAs	Breast	34.87 ± 0.74	31.13 ± 4.54	0.430
	Leg	28.27 ± 1.07	23.85 ± 2.01	0.073
	P value	0.001	0.164	
∑ UFAs	Breast	75.48 ± 0.55	66.32 ± 4.18	0.047
	Leg	69.78 ± 0.80	65.01 ± 1.96	0.040
	P value	<0.001	0.780	
∑ DFAs	Breast	84.57 ± 0.64	78.33 ± 1.92	0.008
	Leg	81.65 ± 0.55	76.13 ± 1.84	0.012
	P value	0.004	0.423	
∑ OFAs	Breast	13.15 ± 0.28	16.26 ± 1.38	0.045
	Leg	16.68 ± 0.44	18.90 ± 0.85	0.037
	P value	<0.001	0.127	
∑ UFAs / ∑ SFAs	Breast	3.09 ± 0.08	2.19 ± 0.26	0.005
	Leg	2.32 ± 0.09	1.92 ± 0.17	0.063
	P value	<0.001	0.425	
∑ PUFAs / ∑ SFAs	Breast	1.43 ± 0.04	1.06 ± 0.16	0.049
	Leg	0.94 ± 0.05	0.71 ± 0.09	0.059
	P value	<0.001	0.093	
TI	Breast	0.16 ± 0.00	0.42 ± 0.25	0.308
	Leg	0.23 ± 0.01	0.29 ± 0.02	0.082
	P value	<0.001	0.618	
AI	Breast	0.21 ± 0.00	0.50 ± 0.18	0.127
	Leg	0.29 ± 0.01	0.47 ± 0.06	0.018
	P value	<0.001	0.883	
NVI	Breast	2.84 ± 0.05	2.87 ± 0.50	0.949
	Leg	2.82 ± 0.07	2.68 ± 0.07	0.238
	P value	0.820	0.713	

SFAs: saturated fatty acids. MUFAs: monounsaturated fatty acids. PUFAs: polyunsaturated fatty acids. UFAs: unsaturated fatty acids. DFAs: desirable fatty acids. OFAs: odd fatty acids. TI: thrombogenic index. AI: atherogenic index. NVI: nutritive value index.

### 3.5 Sensory attributes

The results of the sensory attributes of breast and leg meat from native Mexican guajolotes are presented in Table 6. The panelists evaluated the chewiness of breast meat from males, with higher ( P<0.05 ) scores than breast meat from females. In both sexes, color intensity of leg meat had higher ( P<0.001 ) scores than in breast meat. Aroma, flavor, tenderness, juiciness, and overall acceptance were not influenced by sex or muscle type ( P>0.05 ).

**Table 6:** Means ( ± standard error) of sensory attributes in native Mexican guajolote meat as influenced by sex and muscle type.

Item	Muscle	Sex		P value
		Male	Female	
Aroma	Breast	5.30 ± 0.29	4.93 ± 0.37	0.444
	Leg	5.13 ± 0.30	4.83 ± 0.29	0.484
	P value	0.696	0.833	
Flavor	Breast	5.13 ± 0.27	4.66 ± 0.37	0.322
	Leg	4.53 ± 0.42	4.40 ± 0.34	0.809
	P value	0.244	0.606	
Tenderness	Breast	5.53 ± 0.27	5.00 ± 0.27	0.181
	Leg	4.80 ± 0.39	4.93 ± 0.34	0.800
	P value	0.136	0.881	
Chewiness	Breast	6.00 ± 0.29	4.96 ± 0.31	0.023
	Leg	5.06 ± 0.39	5.43 ± 0.38	0.509
	P value	0.068	0.354	
Juiciness	Breast	4.60 ± 0.34	4.66 ± 0.37	0.897
	Leg	5.10 ± 0.32	4.40 ± 0.30	0.127
	P value	0.303	0.585	
Color	Breast	3.80 ± 0.27	3.33 ± 0.37	0.325
	Leg	5.26 ± 0.20	5.00 ± 0.23	0.405
	P value	0.001	0.001	
Overall acceptance	Breast	5.46 ± 0.23	5.00 ± 0.31	0.243
	Leg	5.00 ± 0.27	5.10 ± 0.33	0.817
	P value	0.205	0.827	

## 4 Discussion

### 4.1 Carcass characteristics

The current study compared carcass composition and physicochemical and sensory attributes in breast and leg meat from native Mexican guajolotes as influenced by sex. Slaughter weight and carcass characteristics were affected by sex due to the sexual dimorphism that characterizes most domestic birds (Yamak et al., 2016; Uhlířová et al., 2018; Cygan-Szczegielniak et al., 2019). The sexual size dimorphism of the birds could be attributed to the usual between-sex differential hormonal effects on growth (Ajayi et al., 2012). Growth rates for females tend to plateau at earlier ages than males. These differences in growth patterns result in higher body weights for males and extend also to carcass characteristics (Maynard et al., 2022). Slaughter weight was almost 50 % greater in males than in females, and as expected, the hot and cold carcass weights were higher in males than in females. Also, males had a higher dressing than females. The dressing percentages obtained in male (65.4 %) and female (59.9 %) native guajolotes were lower than the dressing percentages (71.2 % to 82.7 %) reported in turkeys from commercial lines (İşgüzar, 2003; Majumdar et al., 2005; Loyra et al., 2013; Chartrin et al., 2019). These differences are common in poultry due to metabolic rate differences among native and commercial genotypes (Rajkumar et al., 2016; Khan et al., 2019). Likewise, selection progress in meat-type poultry, such as turkeys, has contributed to an increase in their body weight, an improved carcass composition, and a substantial rise in carcass dressing percentage (Murawska, 2017). In contrast, the lack of selection pressure on growth and yield in guajolotes is likely the reason that they are smaller than turkeys. Age, feeding, and environmental factors also might account for this variation.

On the other hand, all carcass part weights exhibited sexual dimorphism and were higher in males than in females. However, the carcass part yields were similar between sexes, except for drumstick and wing yields, which were higher in females than in males. An important sex effect on carcass part weights was also reported by other authors (İşgüzar, 2003; Murawska et al., 2015; Tůmová et al., 2020), who found that male turkeys presented heavier carcass parts than females. This is due to the fact that the turkey is characterized by a strong sexual dimorphism on body weight, resulting in a higher carcass weight and cut pieces in males than in females (Chartrin et al., 2019). Particularly, breast weight difference between males and females is due to the hypertrophy of the muscle fibers and to more and/or longer muscle fibers (Chartrin et al., 2019). Breast constituted the heaviest carcass component followed by back with ribs, thighs, drumsticks, and wings, regardless of the sex. Consistent trends were observed in carcass part yields in both males and females. In meat-type birds, breast and leg weight is an important economic consideration (Murawska et al., 2015). Similar findings were reported in previous studies (İşgüzar, 2003; Majumdar et al., 2005; Murawska et al., 2015), where carcass composition in turkeys from commercial lines is based mainly on breast, back, thigh, and drumstick yields. The cited authors also observed significant age-related changes in tissue composition of carcass parts. According to Murawska et al. (2015), in turkeys, selection for enlarged breast muscles is due to the fact that consumers generally prefer meat of this muscle.

In the present study, heart and liver were heavier in males than in females, while gizzard weight did not vary among both sexes. However, females had higher abdominal fat content than their male counterparts. In this regard, İşgüzar (2003) reported that bronze and white 18-week-old male turkeys showed higher heart and liver weights than females. Another study reported that liver, gizzard, and heart weights were similar in males and females of BUT9 hybrid species in the early part of the growth period, but they diverged from 35 d of age for the gizzard, 56 d for the liver, and 77 d for the heart. However, the allometric coefficients describing the growth of each of these internal organs in relation to the increase in body weight were the same for males and females (Tůmová et al., 2020). Also, Murawska (2017) reported that in BIG 6 turkeys, growth rates of individual organs vary with age. The higher fat content in females than in males could be explained by differences in fat deposition. In addition, it has been observed that in poultry, the fat content in females increases at a faster rate than in males as the birds mature (Tůmová et al., 2020; El-Tarabany et al., 2022). Regarding the non-carcass components, these also varied due to the sexual dimorphism of guajolotes. Neck, head, and feet were heavier in males than in females. Similar findings were also reported in other poultry species (Murawska, 2017; Kokoszyński et al., 2020; Biesek et al., 2021).

### 4.2 Physical characteristics

According to Semwogerere et al. (2019), the physical characteristics of meat are of paramount importance as they determine the functional properties of meat, which are key during meat processing. Also, physical characteristics are the primary determinants of consumers' willingness to purchase the meat. Particularly, meat quality is closely associated with the decrease in muscle pH post mortem, which in turn is related to the glycolytic enzyme's activity immediately after death. The ultimate pH is shaped by the initial muscle glycogen levels and is of importance when considering meat preservation and stability. A high muscle pH affects shelf life and sensorial quality by its undesirable effect on bacterial growth and meat moistness; conversely, a low pH is associated with poor water-holding capacity and meat color (Cygan-Szczegielniak et al., 2019). The pH values (ranging from 5.75–6.00) obtained in this study varied within the pH range accepted for commercial poultry meat (5.7–6.4) (Gálvez et al., 2018). On the one hand, sex did not affect the pH value measured at 24 h post mortem. On the other hand, in females, the pH values differed among muscles types. The highest pH value was observed in leg meat. These pH differences are probably due to the differences in muscle type and glycogen content, which change according to the proportion of the muscle fibers that are responsible for different patterns of muscle metabolism (Khan et al., 2019). The results of the present study are consistent with the study of Gálvez et al. (2018), who found that sex had no significant effect on the pH values of breast and thigh meat in hybrid turkeys. Likewise, breast meat had lower pH values than thigh meat (6.03 vs. 6.29, respectively). On the contrary, Chartrin et al. (2019) reported that male breeder turkeys from the Grademaker line slaughtered at older ages presented a lower pH in breast and thigh muscles than pH measured in females, indicating higher glycogen reserves.

Meat color is another important attribute used to assess the freshness and quality of meat by consumers and is closely related to the ultimate pH (Uhlířová et al., 2018; Cygan-Szczegielniak et al., 2019). In the present study, breast meat of females was darker (lower $L^{*}$ value) and yellower (high $b^{*}$ value), while leg meat was greener (lower $a^{*}$ value) than that of males. According to Khan et al. (2019), meat color may be influenced by the heme pigments, genetics, and feeding. For example, the consumption of vegetation in the outdoor space could contribute to increased meat yellowness because plant material contains abundant carotenoid pigments (Cygan-Szczegielniak et al., 2019). Moreover, Semwogerere et al. (2019) suggested that meat color variation is also attributed to the effect of water temperature during the defeathering process. Similar to the present study, Sarica et al. (2011) reported that color characteristics of breast meat were different between sexes from different turkey genotypes; the breast meat of females had lower $L^{*}$ values (55.55 vs. 56.12) and higher $a^{*}$ (6.71 vs. 6.37) and $b^{*}$ (1.93 vs. 1.48) values than those of males. In this regard, Gálvez et al. (2018) found that only the $a^{*}$ value was affected by sex in Hybrid turkeys; breast and thigh meat from males was redder than meat from females.

Water-holding capacity is an important attribute of meat quality, and if it is low, meat will lack juiciness. This means that more water could be released during storage and processing of meat, resulting in weight loss in the final product, as well as economic losses (Cygan-Szczegielniak et al., 2019; Onk et al., 2019). Our results showed that male leg meat had higher water-holding capacity than that of females, whereas breast meat from females had higher drip loss values. Specifically, breast meat from both genders was characterized by high values of water-holding capacity when compared to leg meat. In relation to this, Onk et al. (2019) explained that the discrepancy regarding the ability of meat to retain moisture might be attributed to the extent of pH decline post mortem. Sarica et al. (2011) investigated the effects of sex on some meat quality traits of different turkey genotypes and reported that breast meat of females had higher water-holding capacity values than those of the males. Another study on hybrid turkeys reported that males had breast muscles displaying higher drip loss during storage and higher cooking loss, resulting in a lower technological yield than that of females (Chartrin et al., 2019).

### 4.3 Proximate composition

Poultry meat is considered an excellent food for human consumption because of its quality and quantity of protein contents that play a vital role in meat quality assessment (Sabow, 2020). In this study, it was found that sex had a significant effect on the proximate composition of guajolote meat. In particular, breast meat from males had higher moisture (73.95 % vs. 72.98 %) and crude protein contents (24.19 % vs. 22.62 %) but lower ash content (1.09 % vs. 1.12 %) compared to females. In addition, protein and ash contents in breast meat were significantly higher than in leg meat, regardless of sex. However, leg meat from both genders had higher moisture and fat contents. This observation is supported by a study from Majumdar et al. (2005), who demonstrated that, irrespective of the age of the turkeys, breast meat contained more crude protein and less fat than leg meat. According to Gálvez et al. (2018), this could be related to muscle type composition, as leg and thigh meat is formed from several muscles with a higher proportion of red fibers and greater lipid content than breast meat. In this regard, Sarica et al. (2011) found that dry matter, crude protein, and fat contents of breast meat and the crude protein and fat contents of thigh meat were affected by sex in commercial turkeys. Females presented higher contents of dry matter in breast muscle and fat in breast and thigh muscles but lower crude protein in breast and thigh muscles compared to males. On the contrary, Gálvez et al. (2018) reported that sex had no effect on the chemical components of meat from either breast or thigh samples in turkeys. These authors found a mean value of 74.9 % for moisture, whereas that of protein was above 20 %; these values were slightly higher in females than in males, and, also, protein was higher in breast than in thigh samples (24.2 % vs. 20.4 %). Selamat et al. (2022) stated that the higher protein content of village chicken was related to the feed provided and the outdoor system that both contribute to muscle development and led to higher protein. In general, guajolote meat is comparable in terms of proximal composition to the majority of turkey meat that is considered a significant resource of protein.

### 4.4 Fatty acid profile

The fatty acid profile is an important factor determining the quality of animal products (Skiepko et al., 2016). Lipid and fatty acids in muscle are among the major factors that influence meat quality, particularly palatability and nutritional value (Sabow, 2020). In our experiment, when the proportions of individual fatty acids in the breast and leg meat of native guajolotes were considered, arachidonic (C20:0) and erucic acids (C22:1n9) varied according to sex. Also, Gálvez et al. (2018) reported that sex had an effect in 12 out of 25 fatty acids in breast meat and 15 out of 25 fatty acids in thigh meat of commercial turkeys. These results agree with those reported by other authors, who observed differences in meat fatty acid profile between sexes in chickens (Suchy et al., 2016), turkeys (Skiepko et al., 2016), and ducks (Onk et al., 2019). As expected, palmitic (C16:0), oleic (C18:1n9c), and linoleic acids (C18:2n-6c) were the predominant fatty acids found in breast and leg meat from native guajolotes. Similar results regarding predominance order of these fatty acids have also been found in roosters (Amorin et al., 2016), local Polish goose (Wołoszyn et al., 2020), Japanese quail (Sabow, 2020), and broilers and hens (El-Tarabany et al., 2022).

In the current study, the breast and leg meat from native guajolotes was characterized by the prevalence of ∑ MUFAs (35.18–41.51 g per 100 g of fat), followed by ∑ SFAs (24.46–34.95 g per 100 g of fat) and ∑ PUFAs (23.85–34.87 g per 100 g of fat). Females had greater concentrations of ∑ SFAs in the breast and leg muscles as compared to males. However, males had a higher proportion of ∑ MUFAs in breast meat compared to females. The different compositions of MUFAs and PUFAs in the muscle of different growth rates with the same diet may also be due to different dietary habits of the birds (Nur Mahiza et al., 2021). Dal Bosco et al. (2012) suggested that the low MUFA levels observed in chickens from pure breeds reared under organic systems can be attributed to the higher intake of pasture with respect to feed and to the different intramuscular fat content of birds. In the current study, the dominant MUFA in the meat was oleic acid (C18:1n9c). These results are consistent with those obtained previously in commercial Chinese chickens (Chen et al., 2016) and local Polish goose varieties (Wołoszyn et al., 2020). Similarly, Chartrin et al. (2019) demonstrated that sex had effects on the fatty acid composition of turkeys meat; females had a higher SFA content than males. In this regard, Wołoszyn et al. (2020) described that for preventing cardiovascular disease it is advantageous to consume food enriched with MUFAs, which has favorable influence on the blood lipid profile. Palmitic acid (C16:0) was the most abundant SFA (24.46 %–34.95 %), followed mainly by stearic (C18:0) (9.08 %–12.01 %), myristic (C14:0) (0.95 %–3.22 %), and lauric (C12:0) (0.60 %–5.05 %) acids. According to Wołoszyn et al. (2020), these fatty acids occur naturally in all animal fat and are major products of the fatty acid synthase system; accordingly lauric and myristic acids were detected at low concentrations, thus demonstrating a positive factor in their consumption because they promote hypercholesteremia, while stearic acid is neutral in the body as it is directly metabolized into oleic acid (Skiepko et al., 2016).

It was found that breast and leg meat from males contained the highest amount of ∑ UFAs (75.48 and 69.78 g per 100 g of fat, respectively) and ∑ DFAs (84.57 and 81.65 g per 100 g of fat, respectively) and the lowest amount of ∑ OFAs (13.15 and 16.68 g per 100 g of fat, respectively) compared to females. In a study, Gálvez et al. (2018) found that male turkeys presented higher amounts of ∑ SFAs than females in breast and thigh muscles, and these differences were mainly due to males having the highest values of stearic acid (C18:0) and, to a lesser extent, to the values of myristic (C14:0) and heptadecanoic (C17:0) acids. The UFAs are classified as essential, meaning that the organism is unable to generate them, and therefore they must be provided in the feed. These substances exert significant effects on many aspects of organism health. They favorably affect prognosis in cardiovascular diseases; are highly beneficial for the brain and quality of vision; and, in addition, strengthen immunity and help to cure eczema, acne, and psoriasis (Suchy et al., 2016). Therefore, poultry meat with high UFA content is preferable for customers due to its low cholesterol (hypercholesterolemic index) and lower atherogenic index (Attia et al., 2017). In relation to this, Nur Mahiza et al. (2021) affirmed that slow-growing birds, as village chickens, might be better sources of desirable fatty acids than the commercial broiler.

The ∑ UFA / ∑ SFA and ∑ PUFA / ∑ SFA ratios are commonly used parameters to judge meat nutritional value and healthiness of intramuscular fat for human consumption. In general, a ratio of ∑ PUFA / ∑ SFA greater than 0.45 is recommended in human diets to prevent the development of cardiovascular diseases and some chronic diseases (Wołoszyn et al., 2020). In this study, ∑ UFA / ∑ SFA and ∑ PUFA / ∑ SFA ratios ranged from 1.92 to 3.09 and 0.71 to 1.43, respectively, and were significant higher in breast meat of males than of females. In general, the ∑ PUFA / ∑ SFA ratios found were consistent with the recommended values, which indicate improved balance of fatty acids in analyzed tissues (Wołoszyn et al., 2020). The values obtained in the present study for ∑ UFA / ∑ SFA and ∑ PUFA / ∑ SFA ratios were consistent with those found in duck (Onk et al., 2019), laying hens (Semwogerere et al., 2019), and local Polish goose (Wołoszyn et al., 2020).

On the other hand, for a better understanding and nutritional evaluation of fat, the use of health indices based on the functional effects of the fatty acids is essential. The thrombogenic (TI) and atherogenic (AI) indexes should be maintained as low as possible in a healthy heart diet (Semwogerere et al., 2019). Thus, the smaller the TI and AI values, the greater the protective potential for coronary artery disease. In terms of human health, the TI and AI, which are less than 0.5 and 1.0, respectively, in the diet, are recommended (Wołoszyn et al., 2020). The TI, AI, and NVI obtained in the present study ranged from 0.16–0.42, 0.21–0.50, and 2.68–2.87, respectively, but did not significantly differ between sexes, except the AI that had a higher value in the leg meat of females than males. The TI and AI values found in the present study were lower than those obtained by Gálvez et al. (2018), who described values of TI = 0.89–0.88 and 0.98–0.92 and AI = 0.43–0.43 and 0.46–0.45 in breast and thigh muscles from commercial turkeys, respectively. They also reported that females presented the best TI values in thigh samples (0.92 vs. 0.95, for females and males, respectively). Similar values have been reported by Semwogerere et al. (2019) in breast meat from laying hens (TI = 0.60–0.80 and AI = 0.40–0.50), by Onk et al. (2019) in breast meat from ducks (TI = 0.34–0.36, AI = 0.29–0.31, and NVI = 2.38–2.61), and by Wołoszyn et al. (2020) in breast muscles from local Polish goose (TI = 0.66–0.74, AI = 0.36–0.37, and NVI = 1.88–2.17). In general, the breast and leg meat of native guajolote studied in the present work showed TI and AI lower than the recommended values; therefore, this is very desirable from a human health point of view.

### 4.5 Sensory attributes

Sensory evaluation is a useful tool for quality assessment of the various foods, such as meat (Uhlířová et al., 2018). Flavor is a combination of taste and aroma and, together with texture, forms the core of the sensory profile of meat and meat products. The aforementioned attributes are correlated to the physicochemical characteristics of meat and meat products (Semwogerere et al., 2019). In the current study, the mean score for all sensory attributes evaluated varied between values of 3 and 6 for both sexes and muscle types of native guajolotes. However, aroma, flavor, tenderness, juiciness, and overall acceptance of meat were not influenced by the evaluated factors. On the other hand, the breast meat of males received a higher chewiness score than that of females. Also, according to the panelist evaluations, leg meat of both sexes was judged to be more colored than that of breast meat. This can be explained by the intense color of red muscle fibers in contrast with the whitish fat of breast meat (Remm et al., 2011). In addition, it is known that the thigh meat color may also be influenced by species, diet, and exercise of animals (Khan et al., 2019). These findings are confirmed by Chartrin et al. (2019), who observed that turkey male thighs were judged to be more colored, juicier, and stringier than those of females, whereas male breasts were less tender, stringier, and less sticky than the breasts of females. Their global flavor was lower, and they were less appreciated than those of females.

## 5 Conclusions

In conclusion, sex had an effect on some carcass traits. Males had higher carcass weights, dressing percentage, and carcass part weights than females. Moreover, the quality of breast and leg meat varied between sexes. Breast meat from males was characterized by higher lightness, water-holding capacity, moisture content, crude protein, MUFAs, UFAs, DFAs, UFA / SFA ratio, PUFA / SFA ratio, and chewiness scores. Thus, from a nutritional point of view, the meat from male guajolotes was preferable to that from females. Therefore, guajolote meat is a healthy food that can be ideally incorporated into the human diet.

## Data Availability

The original data from the paper are available from the corresponding author upon reasonable request.
